# A spiked human proteomic dataset from human osteogenic differentiated BMSCs and ASCs for use as a spectral library, for modelling pathways as well as protein mapping

**DOI:** 10.1016/j.dib.2019.104748

**Published:** 2019-11-05

**Authors:** Mehran Dadras, Katrin Marcus, Johannes Maximilian Wagner, Christoph Wallner, Mustafa Becerikli, Henriette Jaurich, Stephanie Dittfeld, Marcus Lehnhardt, Bettina Serschnitzki, Annika Guntermann, Lukas Schilde, Björn Behr, Caroline May

**Affiliations:** a Medizinisches Proteom-Center, Ruhr-University Bochum, Bochum, Germany; b BG University Hospital Bergmannsheil, Department of Plastic Surgery, Bochum, Germany

**Keywords:** Proteome, Spectral peptide library, Mesenchymal stromal cells, Adipose derived stromal cells, Bone marrow derived stromal cells, Osteogenic differentiation, Osteogenesis

## Abstract

This article describes a mass spectrometry data set generated from osteogenic differentiated bone marrow stromal cells (BMSCs) and adipose tissue derived stromal cells (ASCs) of a 24-year old healthy donor. Before osteogenic differentiation and performing mass spectrometric measurements cells have been characterized as mesenchymal stromal cells via FACS-analysis positive for CD90 and CD105 and negative for CD14, CD34, CD45 and CD11b and tri-lineage differentiation. After osteogenic differentiation, both cell types were homogenized and then fractionated by SDS gel electrophoresis, resulting in 12 fractions. The proteins underwent an in-gel digestion, spiked with iRT peptides and analysed by nanoHPLC-ESI-MS/MS, resulting in 24 data files. The data files generated from the described workflow are hosted in the public repository ProteomeXchange with identifier PXD015026. The presented data set can be used as a spectral library for analysis of key proteins in the context of osteogenic differentiation of mesenchymal stromal cells for regenerative applications. Moreover, these data can be used to perform comparative proteomic analysis of different mesenchymal stromal cells or stem cells upon osteogenic differentiation. In addition, these data can also be used to determine the optimal settings for measuring proteins and peptides of interest.

**Table:** Specifications Table

Subject area	*Proteomics*
More specific subject area	*Mass spectrometric data set generated from osteogenic differentiated bone marrow stromal cells (BMSCs) and adipose tissue derived stromal cells (ASCs).*
Type of data	*Raw files, mgf files and msf files*
How data was acquired	*Mass spectrometry (Q Exactive HF mass spectrometer operated in data-dependent acquisition (DDA) mode performing HCD fragmentation)*
Data format	*Raw, unfiltered*
Experimental factors	*Data were obtained by mass spectrometric DDA measurements of human* osteogenic differentiated BMSCs and ASCs *spiked with iRT peptides for generation of a spectral library*
Experimental features	Osteogenic differentiated BMSCs and ASCs *were fractionated by SDS gel electrophoresis and in-gel digested, resulting in 12 fractions of each cell type.*
Data source location	*Bochum, Germany (51°26′43.4″N 7°15′27.9″E)*
Data accessibility	*The data files are hosted in the public repository ProteomeXchange with identifier PXD015026. (*http://proteomecentral.proteomexchange.org/cgi/GetDataset?ID=PXD015026*)*

**Table:** 

**Value of the Data** • This data set can be taken as a spectral library for osteogenic differentiated mesenchymal stromal cells, for example, in the context of regenerative medicine and bone tissue engineering. • It can be used for data independent acquisition (DIA) based proteomic analysis of stromal/stem cells in the context of osteogenic differentiation, for example, to identify key proteins in a comparison of cells of different origin. • The data set can be also used to map proteins. • The data set can be used to optimize parameters for the identification of proteins/peptides of interest in mesenchymal stromal cells. • The data set can be used to model pathways located in mesenchymal stromal cells of bone and fat tissue undergoing osteogenic differentiation.

## 1 Data

**Fig. 1:**
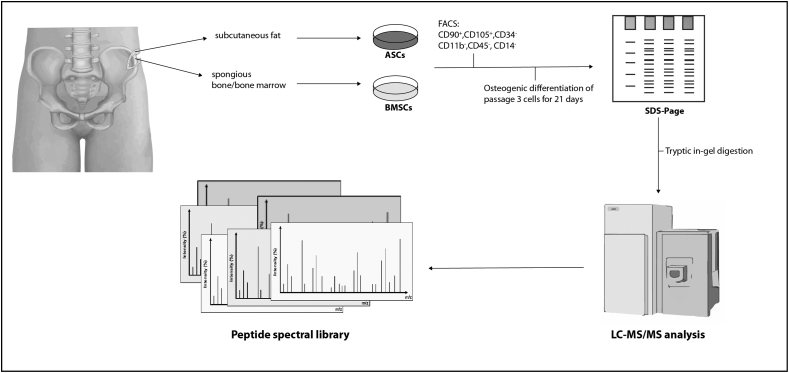
This illustration shows the workflow overview. BMSCs and ASCs derived from subcutaneous fat and spongious bone were isolated, expanded, characterized and then underwent osteogenic differentiation in passage 3. Both cell types were homogenised and then fractionated by SDS gel electrophoresis. The proteins underwent an in-gel digestion with trypsin, spiked with iRT peptides and measured by a data-dependent mass spectrometric approach (DDA).

The dataset contains raw mass spectrometry proteomic data of human osteogenic differentiated BMSCs and ASCs derived from subcutaneous fat and spongious bone of a 24-year old healthy donor. The samples were spiked with iRT peptides and the library was created by proteomic analysis in DDA mode. The dataset consists of 12 raw files per cell type for a total of 24 files with a mean of 46566.75 MS/MS spectra (17976.875 PSMs) per file. Fig. 1 describes the workflow used to acquire the data. The datafiles were deposited at ProteomeXchange database with identifier PXD015026.

## 2 Experimental design, materials and methods

### 2.1 Human primary cell culture

Specimen from subcutaneous fat and spongious iliac bone of a healthy, non-smoking, 24-year-old male patient were taken during an autologous bone transplantation procedure. Cell isolation was performed according to modified standard protocols [1,2]. Adipose tissue was washed four times with prewarmed DPBS (PAN Biotech, Germany) and transferred onto a petri dish. Blood vessels and connective tissue were carefully removed before the tissue was minced into small pieces using sterile scissors. The tissue sample was transferred into a 50 mL falcon tube and weighed. 5 mL of collagenase IV (1 mg/ml, Cell Systems, Germany) per gram of tissue material was used for digestion. The falcon tube was placed on a heated shaker and incubated for 1 hour at 37 °C and 330 rpm. Thereafter, the reaction was stopped by adding 25 mL of base medium (DMEM/HAM's F-12 [PAN Biotech, Germany], 10% fetal bovine serum, 1% Penicillin/Streptomycin). Spongious bone was washed multiple times with base medium, the wash fractions were collected and combined.

Cell suspensions of each source were then filtered through a 100 μm cell strainer and centrifuged at 1400 rpm for 5 min at room temperature (Megafuge 1.0, Heraeus, Germany). The cell sediment was resuspended in cold red blood cell (RBC) lysis buffer (900 mL Milli-Q-water, 8 g ammonium chloride, 0.8 g sodium hydrogen carbonate, 0.4 g EDTA) and incubated on ice. The lysis was stopped after 8 min by addition of base medium. The cells were centrifuged at 1400 rpm for 5 min at room temperature. Cell sediment was resuspended in base medium and cell concentration was measured using the CASY® Cell Counter (OLS OMNI Life Science, Germany). Afterwards, cells were then plated in cell culture flasks according to their amount (10^6^ cells per T-75 flask) and maintained in an incubator at 37 °C and 5% CO_2_. 48 hours after plating, non-adherent cells were washed off with prewarmed DPBS (PAN Biotech, Germany). Fresh base medium was added and changed thereafter every 2–3 days.

Cells of passage three were used for osteogenic differentiation. For this, cells were washed and detached using 0.05% Trypsin/EDTA (Trypsin: PAA Laboratories, Germany; EDTA: Sigma-Aldrich, Germany). Cell suspension was centrifuged at 1400 rpm for 5 min at room temperature and cell sediment was resuspended in base medium. Cells were plated in a density of 2 × 10^5^ cells per 10 cm culture dish. Then, the osteogenic differentiation was induced by culturing the cells in the osteogenic differentiation medium consisting of the base medium, 10 mM β-glycerophosphate, 10 nM dexamethasone and 250 μM ascorbic acid for 21 days. Afterwards, the cells were washed and detached using a cell scraper in presence of DPBS. Cells were collected and centrifuged at 900 rpm for 5 min at 4 °C. Supernatant was aspirated and the samples were stored at −80 °C.

### 2.2 Sample preparation

Homogenization of the BMSC as well as the ASC sample was performed as described previously [3]. In short, per 100 mg cells, 400 μL precooled RIPA buffer (Cell Signaling Technology Europe, Germany) was added as well as 0.35 g glass beads (Ø 1.25–1.65 nm; Ø 0.25–0.5 nm; Carl Roth, Germany). Cell samples were homogenized by sonication (Potter S. Homogenizer, B. Braun, Germany), supernatants transferred to a new reaction tube and again sonicated in an ice-cold ultrasonic bath (BANDELIN electronic GmbH & Co. KG, Germany) for 10 sec for a total of six times. For discarding insoluble fragments samples were centrifuged at 16.000 g for 15 min at 4 °C (centrifuge 5415R, Eppendorf GmbH, Germany), supernatant transferred to a new reaction tube, aliquoted and stored at −80 °C. Protein concentration was determined by Bradford assay (Sigma-Aldrich, Germany) according to the manufacturer's instructions. Before gel electrophoresis 40 μg cell lysate were displaced with 4x concentrated LDS sample buffer (pH 8.5, 26.5 mM Tris HCl, 35.25 mM Tris base, 2% LDS, 10% glycerol, 0.055 mM Coomassie blue G250 and 0.045 mM phenol red) and vortexed. To reduce disulphide bridges and denaturation, 2 mM DTT was added and the samples incubated for 10 min at 350 rpm and 90 °C in a thermomixer (Thermomixer comfort, Eppendorf GmbH, Germany). To remove insoluble components, the sample was again centrifuged for 4 min at 5.000 g at room temperature.

### 2.3 Complete SDS gel electrophoresis

Gel electrophoresis was performed as described by Guntermann et al. [4]. For this, 60 μg of protein per lane was loaded on a NuPAGE™ 4–12% Bis-tris gel (Fisher Scientific, Germany).

### 2.4 Gel staining

Gels were stained with Coomassie blue (SimpleBlue™ SafeStain, Thermo Fisher Scientific, USA) according to the manufacturer's instructions.

### 2.5 Trypsin in-gel digestion and peptide extraction

Lanes on the NuPAGE™ 4–12% Bis-tris gel were cut into 12 single bands and every single band transferred into a glass vial. The in-gel digestion and peptide extraction were performed as described previously by Guntermann et al. [4]. In short, gel pieces were incubated for 10 min in 50 mM ammonium bicarbonate (solution A) (Sigma-Aldrich) followed by incubation with 50% (v/v) 50 mM ammonium bicarbonate with 50% (v/v) 100% acetonitrile (solution B) (Merck KGaA, Germany). Gel pieces were again incubated in solution A, the ammonium bicarbonate buffer discarded, each glass vial filled with 50 μL of 10 mM dithiothreitol (AppliChem GmbH, Germany), followed by incubation for 1 hour at 350 rpm and 56 °C. Afterwards the dithiothreitol solution was discarded and each glass vial filled with 50 μL of 50 mM iodoacetamide (Merck KGaA, Germany). The gel pieces were incubated for 45 min at room temperature in the dark, then iodoacetamide solution was discarded, and the destaining protocol continued with solution B for 10 min. At last, gel pieces were again washed in solution A for 10 min, afterwards in solution B for 10 min followed by discarding solution B.

For peptide extraction gel pieces were dried in a vacuum concentrator (RVC2-25CD plus, Martin Christ Gefriertrocknungsanlagen, Germany), resuspended in 6 μL of trypsin solution (0.012 μg/μL, Promega Corp., Germany) and incubated overnight. The trypsin digest was stopped, and peptides eluted by incubation for 15 min in 30 μL of a 1:1 solution containing 100% acetonitrile and 0.1% (v/v) trifluoroacetic acid (Merck KGaA, Germany) in an ice-cold ultrasonic bath. This step was repeated for in total two times.

From the resulting peptide extract 4 μL were transferred to a new glass vial, dried in a vacuum concentrator (Eppendorf GmbH) followed by resuspending in 15 μL of 0.1% (v/v) trifluoroacetic acid. Afterwards, one injection volume of iRT peptides (Biognosys, Switzerland) was added.

### 2.6 nanoHPLC settings and mass spectrometry settings

NanoHPLC settings as well as mass spectrometry settings were as described by Guntermann et al. [4]. The solvent gradient profile for the elution of peptides is shown in Table 1.

**Table 1:** Solvent gradient profile for the elution of peptides.

time (min)	% solvent B
0	5
7	5
15	8
110	25
139	40
140	95
146	5
150	5

## Conflict of Interest

The authors declare that they have no known competing financial interests or personal relationships that could have appeared to influence the work reported in this paper.
